# Diabetic retinopathy and pregnancy: an overview of the predictive risk factors for progressive worsening of eye disease during the antenatal period

**DOI:** 10.1038/s41433-025-03677-6

**Published:** 2025-02-14

**Authors:** Maria S. Varughese, Sarita Jacob

**Affiliations:** 1 University of Sheffield Medical School, Royal Hallamshire Hospital, Sheffield, UK; 2 University Hospitals Birmingham NHS Foundation Trust, Birmingham, UK; 3 Institute of Clinical Sciences, University of Birmingham, Birmingham, UK; 4 College of Life and Health Sciences, Aston University, Birmingham, UK; 5 Birmingham, Solihull and Black Country Diabetic Eye Screening Programme, Birmingham, UK

**Keywords:** Risk factors, Public health

Diabetic retinopathy (DR) in pregnancy is a growing problem [1], and more recently there has been a renewed interest for an urgent need to identify patients, who are most vulnerable for worsening DR from the at-risk cohort of predictive co-existing factors [2–7]. Pregnancy in itself, is one of the independent risk factors for the development *and* progression of DR [2]. Although from a practical perspective, both type 1 diabetes mellitus (T1DM) and type 2 diabetes mellitus (T2DM) are risk factors for progression of DR, T1DM is more often associated with new onset DR in pregnancy [1, 2]. The degree of DR at conception, presence of hypertension and pre-eclampsia, level of glycaemic control at the beginning of pregnancy and obesity are noted to be strong risk factors for progression of DR, in conjunction with other aspects like the duration of diabetes, presence of associated diabetic nephropathy, perinatal undernutrition and smoking status [2].

T1DM and T2DM during pregnancy as well as gestational diabetes mellitus (GDM) could possibly lead to the development of severe DR during pregnancy or worsen pre-existing DR [3], and in addition GDM increases the subsequent risk of diabetes for both the mother and the foetus in the future. Therefore, understanding the prevalence, assessing the predictive risk factors contributing to the pathogenesis of DR, as well as evaluating and identifying treatment challenges related to DR in pregnancy, most certainly warrants a cohesive and concerted effort by all specialists involved in pre-conception, antenatal as well as post-natal care [1–3].

DR is the leading cause of blindness in women of reproductive age and pregnancy per se is a known risk factor for the development and progression of DR [2, 4]. About 1 in 12 pregnant individuals with pre-existing diabetes without retinopathy are expected to develop some form of DR during their pregnancy [3, 4]. It is well recognised that if detected and managed early, irreversible vision loss could be effectively prevented in a significant majority of these patients. The presence of DR at first trimester screening is a strong predictor of progression of retinopathy during pregnancy, and in the absence of DR at baseline, progression to more severe forms of DR are uncommon and often do not require treatment [4], allowing for less frequent retinal screening for select patients based on predictive risk [4, 5].

Of late, it has been demonstrated that nearly 25% of women had DR progression from conception through to 12-months postpartum; with almost half of these patients developing sight-threatening disease [6]. DR progression occurring during pregnancy in this recent study was found to predominantly remain unchanged, or worsen after delivery, with very few eyes spontaneously improving postpartum which is a significant cause for concern. Long-term studies have reiterated the fact that managing diabetes during pregnancy can be challenging for the clinician [7–15]. It is therefore prudent to provide pre-conception counselling for women planning pregnancy, and aim for optimum HbA1c target, as it would not only lead to tight glucose control pregestation [8], but it will also prevent a rapid decrease in HbA1c, especially between conception and the third trimester during the antenatal period. This would certainly also help to mitigate the risk of paradoxical worsening of DR related to the magnitude of improvement in HbA1c with sudden, intensive glucose control [5, 8].

It is also pertinent to note that irrespective of ethnicity, the risk of women with no DR in early pregnancy developing referable retinopathy during pregnancy is minimal, and in those patients that do progress, they are unlikely to require treatment [9]. For women with pre-existing DR in early pregnancy, the risk of disease progression during pregnancy is significant [4]. Hence, it is imperative to continue to stratify and manage risk factors to help provide a clearer clinical picture to assist both clinicians and patients in reducing the risk to both the mother and foetus [10, 11]. In particular, the importance of exploring the impact of well-known indicative risk factors for the *development and progression* of ocular complications and how they corelate and apply to smaller populations and individual patients becomes all the more relevant [11].

Systematic reviews and meta-analysis suggest that women with T1DM and T2DM had a comparable risk of DR progression during pregnancy [1, 12]. DR *progression* in pregnant women with diabetes continues to be *higher* than in the non-pregnant population with diabetes [13]. The *prevalence* of DR in pregnant women has been demonstrated to be *similar* in comparison to the non-pregnant diabetic population, however in some studies 1 in 9 participants had sight-threatening DR (STDR) during pregnancy, highlighting the need to optimise DR management in pregnancy given the significant risk of loss of vision [14].

Prioritising the relevance of eye screening intervals in DR especially in pregnancy is crucial [15–17], and educating women with diabetes in the reproductive age group during pre-conception counselling on the importance of adhering to screening recommendations is vital [15, 17]. Pregnancy, delivery and neonatal outcomes among women with DR is reliant on various factors of planned care as they have a higher risk of pregnancy induced hypertension, pre-eclampsia and caesarean section [18–20].

In addition to pregnancy itself being a risk factor for *progression* of DR [2, 21], the additive impact of various physiological changes involving (a) metabolic, (b) vascular, (c) immunologic, and (d) hormonal fluctuations that occur during pregnancy can cause *development* as well as *worsening* of DR [21], and this can eventually lead to permanent visual loss if not addressed with swift interventions. Significant alterations can occur to the eye during pregnancy, highlighting the need for further research to develop clear recommendations that warrants the need to preserve the maternal sight, and also foetal health [21–24].

The need for pre-conception planning and fostering a collaborative approach between members of the multi-disciplinary team involving the midwife, obstetrician, physician, ophthalmologist and paediatrician is indeed paramount [23], given the complex relationship encompassing pregnancy and eye changes [24].

There are still ongoing challenges to address the impact of diabetes mellitus on pregnant women. Despite widespread agreement on the need for rigorous retinal screening for women with pregestational diabetes, things remain far from ideal and more needs to be done towards streamlining global efforts to address pregnant women’s retinal health in the setting of pregestational and antenatal diabetes care [25].
